# Efficient Interfacial Charge Transfer Based on 2D/2D Heterojunctions of Fe-C_3_N_4_/Ti_3_C_2_ for Improving the Photocatalytic Degradation of Antibiotics

**DOI:** 10.3389/fchem.2022.865847

**Published:** 2022-05-23

**Authors:** Zhaohui Huo, Yanmin Liao, Yongyi He, Yifan Zhang, Xiaolin Liao, Qitong Zhang, Haojie Wu, Junjie Shi, Genglong Wen, Haixia Su, Suyang Yao

**Affiliations:** ^1^ School of Chemistry and Materials Science, Guangdong University of Education, Guangzhou, China; ^2^ Engineering Technology Development Center of Advanced Materials & Energy Saving and Emission Reduction in Guangdong Colleges and Universities, Guangzhou, China

**Keywords:** g-C_3_N_4_, Ti_3_C_2_, Fe, 2D/2D heterojunction, photocatalytic degradation

## Abstract

Graphitic carbon nitride (g-C_3_N_4_) has shown to be a promising photocatalyst that, however, suffers from strong charge recombination and poor conductivity, while MXenes have shown to be perfect cocatalysts for the photocatalytic process but show poor stability. In this study, we successfully constructed 2D/2D heterojunctions of Fe-C_3_N_4_/Ti_3_C_2_ for the photocatalytic degradation of antibiotics. In this study, multilayer Ti_3_C_2_ was obtained by etching Ti_3_AlC_2_, and then Fe-C_3_N_4_/Ti_3_C_2_ photocatalyst was prepared by the one-pot microwave method and high-temperature calcination method. The synthesized samples were characterized by XRD, SEM, TEM, XPS, TGA, BET, DRS, PL, and other means. The photocatalytic degradation of tetracycline hydrochloride by Fe-C_3_N_4_/Ti_3_C_2_ was in accordance with the first-order reaction kinetics model, and the apparent rate constant k was 2.83, 2.06, and 1.77 times that of g-C_3_N_4_, Fe-C_3_N_4_, and g-C_3_N_4_/Ti_3_C_2_, respectively. Through the mechanism study, it was shown that the most active species in the reaction system was • O_2_^−^, while h^+^ and •OH had a relatively lower effect on the degradation system.

## Introduction

With the rapid development of modern industry, the problems of environmental pollution and energy shortage are increasingly prominent. Water pollution is particularly prominent among many environmental pollution problems, and antibiotic wastewater is recognized as organic wastewater, that is, difficult to treat (Lindberg et al., 2005). Tetracycline antibiotics are widely used in clinics and belong to broad-spectrum antibiotics. However, tetracycline drugs cannot be completely absorbed after being ingested by organisms, and most of them are discharged in their original form or metabolites (Gibson and Skett, 1986). Moreover, the production process of this class of antibiotics is complex, and a large number of them remain in pharmaceutical wastewater. Tetracycline antibiotics can damage the aquatic environment and cause chronic effects on the behavior, reproduction, and growth of organisms. At the same time, these antibiotics have bactericidal and bacteriostatic effects, resulting in the disappearance of some microbial populations and ecological function damage, which may lead to changes in methane generation, sulfate reduction, nitrogen transformation, and organic matter degradation, bringing great threats to the ecosystem and human health (Pei et al., 2020). At present, the treatment of tetracycline antibiotic wastewater mainly includes the biological method (Xu et al., 2017b), physical method (Wei et al., 2019), and chemical method (Lee et al., 2011), but these methods have disadvantages such as low degradation rate, complex technological process, and high cost, and may produce some new pollutants. Therefore, the development of a green, efficient, and low energy consumption antibiotic degradation technology has become an urgent need. Semiconductor photocatalytic technology can convert renewable solar energy into chemical energy under mild conditions, promote the REDOX reaction, and degrade antibiotics into nontoxic small molecules, so it has become a research hotspot. The core of photocatalytic technology lies in semiconductor materials. At present, a large number of semiconductor materials have been explored and applied in this technology, such as titanium dioxide (TiO_2_) (Gao et al., 2020; Galeano et al., 2019), black phosphorus (P) (Bian et al., 2020; Liu D. et al., 2020), tungsten trioxide (WO_3_) (Fu et al., 2019; Sun et al., 2019), and graphitic carbon nitride (g-C_3_N_4_) (Tong et al., 2017). At present, there are two main aspects that restrict the photocatalytic effect of semiconductors (Liu N. et al., 2018): 1) Low utilization rate of light. Most semiconductor photocatalysts can only be excited by ultraviolet light, which greatly limits the utilization rate of materials to sunlight; 2) the single-component semiconductor materials often have the defect of a high recombination rate of electron–hole pairs, which seriously restricts the photocatalytic effect. Therefore, we need to develop efficient photocatalytic materials with high visible light response and low carrier recombination rate, so as to promote the application of photocatalytic technology. In numerous semiconductor photocatalysts, the nonmetallic photocatalyst g-C_3_N_4_ stands out because of its visible light response, good chemical stability, suitable conduction band (CB) and valence band (VB) location, low cost, and nontoxic advantages; is considered as a metal-free photocatalyst with broad prospects; and has a great research value in the field of photocatalytic treatment of water pollution and hydrolysis of H_2_. However, because of its poor conductivity, high carrier recombination rate, and small specific surface area (Xu et al., 2017a), the practical application of g-C_3_N_4_ is limited. Therefore, researchers modified g-C_3_N_4_ by means of element doping (Li et al., 2018; Zhao et al., 2019), semiconductor recombination (Yang et al., 2020; Yi et al., 2020; Liu et al., 2021), morphology control (Cui et al., 2018), and noble metal deposition (Li et al., 2019), to improve the photocatalytic performance. Among these strategies, doping (metal or non-metal) has been extensively used as a valid method to modulate the energy gap of semiconductors for the treatment of conductive, optical, or other physical properties (Fu et al., 2017; Liu N. et al., 2018). Doping Fe has been recognized as a facile and efficient approach to amending g-C_3_N_4_ (Zheng et al., 2015). g-C_3_N_4_ is rich in N atoms, which are filed with six lone pair electrons (Wang et al., 2009). This unique unit structure is quite appropriate for Fe inclusion. For example, Xu et al., 2019, designed Fe-doped surface alkalization g-C_3_N_4_ photocatalyst, showing good photocatalytic activity, and the degradation rate of tetracycline hydrochloride (TC) within 80 min was 63.70%, which was 1.29 times of that before doping. And, Liu G. et al., 2020, designed CNFe_x_ samples with different Fe doping ratios, and the results showed that when the Fe doping ratio was 0.25, the samples showed the best photocatalytic activity, and the degradation rate of RhB was 87.00% within 60 min, 2.90 times that of pure g-C_3_N_4_. Liu et al. carried out a series of studies about Fe_2_O_3_ (He et al., 2020; Huang et al., 2021a), they have demonstrated that the combination of α-Fe_2_O_3_ can further improve the transfer of photogenerated charges and improved the photoelectric conversion efficiency. In particular, Fe_2_O_3_ combined with g-C_3_N_4_ could improve h^+^ injection efficiency (Huang et al., 2021b). The results show that due to the lower reduction potential of Fe^2+^/Fe^3+^ than that of g-C_3_N_4_, the addition of Fe species can effectively capture the photogenerated carriers of g-C_3_N_4_ and inhibit the recombination of electron–hole pairs. At the same time, the modification of high conductive materials on g-C_3_N_4_ nanosheets to construct heterojunctions is one of the feasible ways to promote charge separation.

MXene is an emerging two-dimensional layered material that shows great potential in the field of photocatalysis due to its superior ability to capture light and metal conductivity. The general formula for MXene is M_n+1_X_n_T_x_, where M represents the early transition metal, X represents carbon or nitrogen, and T_x_ represents the functional groups (such as -OH, -F, and =O) produced in the etching process and attached to the surface of MXene material. In other words, MXene material is a new type of two-dimensional material composed of transition metal carbides, nitrides, and carbonitrides. Among them, Ti_3_C_2_ is a typical MXene material composed of transition metal carbide, which was successfully synthesized for the first time by Naguib et al. (2011). With the advantages of multilayer structure, good electrical conductivity, large specific surface area, and excellent chemical stability, Ti_3_C_2_ has become a research hotspot in the energy field in recent years. At present, a variety of hybrid materials based on Ti_3_C_2_ appear in the field of photocatalysis, such as Ti_3_C_2_/Bi_2_WO_6_ (Huang et al., 2020), Ti_3_C_2_/CdLa_2_S_4_ (Cheng et al., 2020), and Ti_3_C_2_/CdS (Yang et al., 2019, Huang et al., 2020) prepared Ti_3_C_2_/Bi_2_WO_6_ composites by electrostatic assembly method, showing excellent performance in photodegradation of formaldehyde and acetone, and the degradation rate was 2 times and 6.6 times of pure Bi_2_WO_6_, respectively. Studies have shown that this may be attributed to the strong adsorption of formaldehyde and acetone by Ti_3_C_2_ and the effective promotion of photogenerated electron–hole recombination of Bi_2_WO_6_ by Ti_3_C_2_. A large number of studies have shown that (Xiao et al., 2016; Anasori et al., 2017) Ti_3_C_2_ can effectively promote the separation of photogenerated electrons and holes by forming heterojunctions between Ti_3_C_2_ and other semiconductors due to its excellent metal-like conductivity. Therefore, Ti_3_C_2_ is widely used as a cocatalyst to improve the photocatalytic performance of semiconductors. Currently, there was also some work on the research of Ti_3_C_2_ and g-C_3_N_4_ composites. For example, Liu S. et al. (2018) prepared Ti_3_C_2_/g-C_3_N_4_ composites by means of evaporative self-assembly. The heterojunction formed between g-C_3_N_4_ and Ti_3_C_2_ effectively inhibited the photogenerated electric–hole pair composite of g-C_3_N_4_, and ciprofloxacin could be completely decomposed within 150 min, which was about 10% higher than that of pure g-C_3_N_4_. Composites show better performance. Liu et al., 2022, designed a 3D/2D g-C_3_N_4_/Ti_3_C_2_ heterojunction and found that benefiting from the 3D interconnected morphology and the incorporation of Ti_3_C_2_ nanosheets, it exhibited high specific surface area and efficient charge transfer. Yi et al., 2020, designed the compound of alkalized g-C_3_N_4_ with less layer Ti_3_C_2_ to prepare Ti_3_C_2_/g-C_3_N_4_ complex, which could effectively remove TC under visible light irradiation, with a yield of 77.0%. The kinetic constant was 1.8 times higher than that of alkalized g-C_3_N_4_, and the photocatalytic performance was effectively enhanced. Liu et al., 2021, prepared Ti_3_C_2_/g-C_3_N_4_ photocatalysts with different mass ratios. The results showed that when the mass ratio was 1%, Ti_3_C_2_/g-C_3_N_4_ had the best degradation performance for levofloxacin. It was 72.0%, which was 2.14 times that of the g-C_3_N_4_ monomer. Yang et al., 2020, prepared the ultrathin Ti_3_C_2_/g-C_3_N_4_ complex by directly calcining the mixture of massive Ti_3_C_2_ and urea, which showed good performance in the photoreduction of CO_2_, and the total CO_2_ conversion rate was 8.1 times higher than that of pure g-C_3_N_4_. These results showed that the 2D/2D heterojunction formed by g-C_3_N_4_ and Ti_3_C_2_ could effectively promote the charge separation of g-C_3_N_4_ and improve photocatalytic performance. In view of the disadvantages of g-C_3_N_4_, such as small specific surface area, easy recombination of electron–hole pairs, and low visible light utilization, the peeling process of Ti_3_C_2_ reported at present was complicated and had certain risks, and the “accordion” lamellar structure is unstable and easy to recombine.

In this study , Fe-C_3_N_4_/Ti_3_C_2_ was prepared by the one-pot microwave method and high-temperature calcination method. The photocatalytic performance of g-C_3_N_4_ was improved by the Fe doping and Ti_3_C_2_ composite, and TC was used as the target degradation material to investigate the photocatalytic performance of the sample and explore the photodegradation mechanism. The experimental results showed that both Fe doping and Ti_3_C_2_ composite could reduce the bandgap energy of g-C_3_N_4_ and improve the visible light utilization of the material; at the same time, it could effectively inhibit the recombination of electron–hole pairs of g-C_3_N_4_, improve the quantum efficiency, and enhance the photocatalytic performance. The highlights of this work were as follows: 1) simple preparation method: the synthesis of g-C_3_N_4_, the intercalation and peeling multilayer Ti_3_C_2,_ and the composite of Ti_3_C_2_ and g-C_3_N_4_ were realized simultaneously by the combination of one-pot microwave method and high-temperature calcination method. Specificaly, urea was used as the precursor to prepare g-C_3_N_4_ in this study. NH_3_ was produced in the process of calcination. As a small molecular substance, NH_3_ could intercalate multilayer Ti_3_C_2_, effectively prevent the stacking of Ti_3_C_2_ layers and play a supporting role, promote the expansion of the spacing of multilayer Ti_3_C_2_ layers, and better form a tight 2D/2D interlayer interface with g-C_3_N_4_. The conventional relatively complex synthesis path of synthesizing monomer g-C_3_N_4_ and multilayer Ti_3_C_2_, and then intercalation and peeling multilayer Ti_3_C_2_ with dimethyl sulfoxide, and then recombination was simplified effectively (Li et al., 2020). 2) For the first time, Fe and Ti_3_C_2_ were introduced to modify g-C_3_N_4_, and the reduction potential of Fe^2+^/Fe^3+^ was lower than that of g-C_3_N_4_, to effectively capture the photogenerated carriers of g-C_3_N_4_. At the same time, the 2D/2D heterojunction formed by g-C_3_N_4_ and Ti_3_C_2_ could effectively promote the charge separation of the g-C_3_N_4_ and explored whether there was a synergistic effect between these two modification methods to further promote the photocatalytic performance of the g-C_3_N_4_.

## Experimental Section

### Materials

Urea, p-benzoquinone (BQ), and ethylenediamine tetraacetic acid disodium (EDTA-2Na) were purchased from Tianjin Damao Chemical Reagent Factory. Lithium fluoride (LiF), tetracycline hydrochloride (TC), and isopropanol (IPA) were purchased from Shanghai Aladdin Biochemical Technology Co., Ltd. Iron nitrate [Fe(NO_3_)_3_·9H_2_O] was purchased from Shanghai McLean Biochemical Technology Co., Ltd. Ti_3_AlC_2_ MAX phase was purchased from Hangzhou Namao Technology Co., Ltd. Hydrochloric acid (37% wt.) was purchased from Guangzhou Chemical Reagent Factory. And, all the chemicals are analytically pure except for special marked, and they were directly used without further purification.

### Preparation of Multilayer Ti_3_C_2_

HCl solution (20 ml of 9 mol/L) was added to the Teflon beaker, and 1.0 g of LiF powder was slowly added to the beaker under the action of a magnetic agitator. After it gets fully dissolved, the solution was stirred for 10 min. To avoid overheating, 1.0 g of Ti_3_AlC_2_ powder was added slowly for about 20 min. After continuous stirring at 35°C for 48 h, they were centrifuged at 4,000 rpm for 20 min, washed with 3 mol/L HCl solution several times to remove the residual LiF after reaction, and then washed repeatedly with deionized water until the pH of the supernatant was close to 7. The collected Ti_3_C_2_ precipitate was freeze-dried thoroughly, and the obtained powder was multilayer Ti_3_C_2_ powder.

### Preparation of Fe-C_3_N_4_/Ti_3_C_2_

**SCHEME 1:**
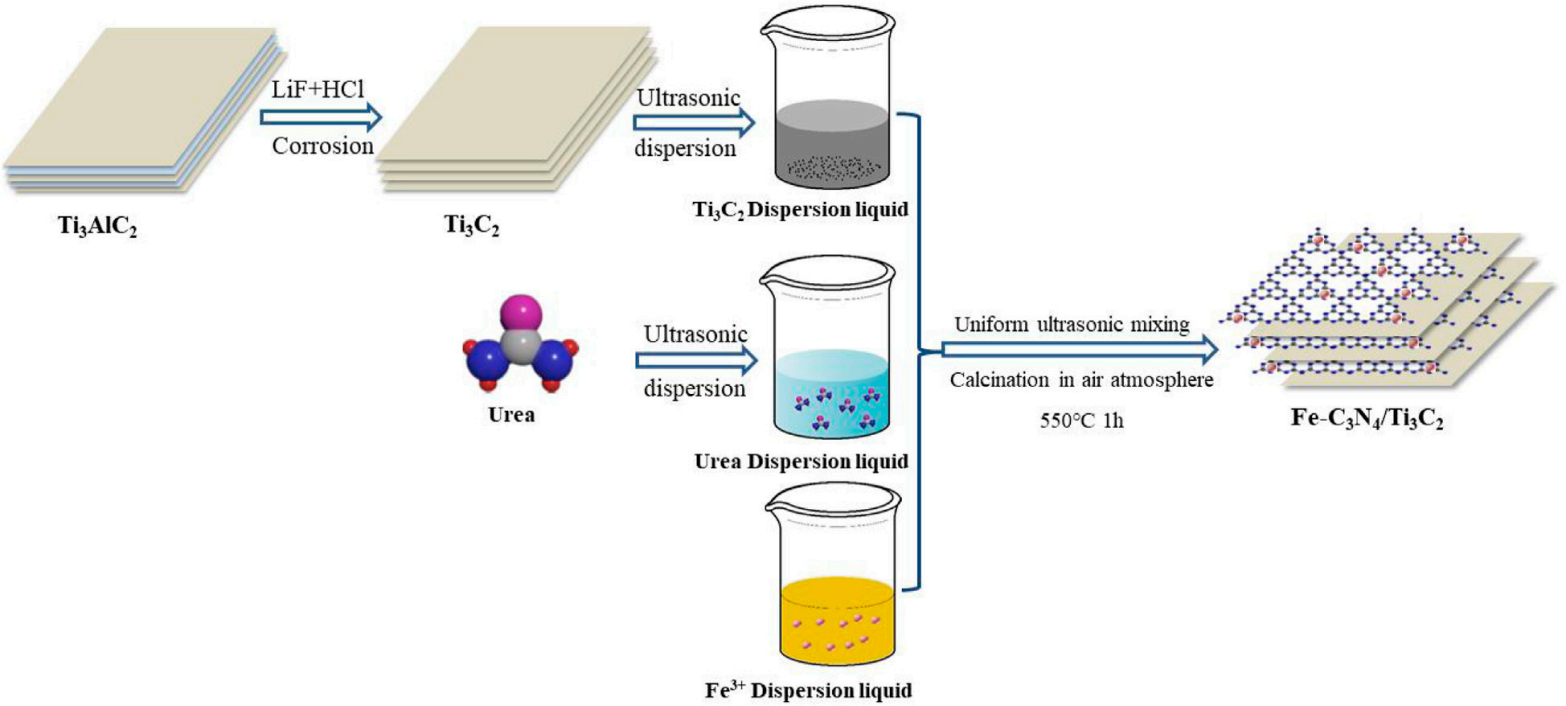
Scheme illustration for Fe-C_3_N_4_/Ti_3_C_2._

Fe(NO_3_)_3_·9H_2_O (0.06 g) was dissolved and dispersed in 50 ml of deionized water, 10 g of urea was added to dissolve completely, and 0.08 g of Ti_3_C_2_ powder was added under an ultrasonic environment to form a uniform dispersion solution, dried thoroughly, and ground evenly. Calcination was carried out in a 50 ml crucible with a lid in a muffle furnace under an air atmosphere. The temperature rose to 550°C at a rate of 50°C/min, and the temperature was held for 1 h. The resulting brown-yellow solid was ground thoroughly to form Fe-C_3_N_4_/Ti_3_C_2_ powder (see Scheme 1).

### Testing and Characterization of Materials

The phase characterization of the samples was determined by an X-ray powder diffract graph (D8 Advance, Brock AXS, Germany), using Cu-Kɑ rays with a wavelength of 154 p.m. and a scanning range of 2θ = 5°∼80°; the morphology and structure of the samples were characterized by MIRA 3 LMU scanning electron microscope SEM (TESCAN Brno, S.R.O., Czech Republic) at a voltage of 15 kV; the morphology and microstructure were also observed by transmission electron microscopy TEM and high-resolution transmission electron microscopy HR-TEM (FEI Talos F200x G2,United States); the element was mapped by the energy-dispersive spectroscopy EDS (FEI Talos F200x G2, Super-X, United States); the element composition and existence form of the samples were determined by X-ray photoelectron spectroscopy XPS (Thermo Scientific K-Alpha Hangzhou Yanqu Information Technology Co., LTD) using the Al-Kα rays; the thermal stability of the samples was characterized by using a thermogravimetric analyzer TGA (TGA-4000, Platinum Elmer, United States); the specific surface area of the samples was analyzed by an automatic specific surface adsorption instrument BET (BELSORP-max, DKSH Commercial Co., LTD., Japan); the optical absorption performance of the sample was reflected by using the UV-visible diffuse reflector DRS (Shimadzu UV-2600, Japan) and calculating the bandgap of the photocatalyst. BaSO_4_ was used as the reference sample; the photocatalytic activity of the samples was analyzed by fluorescence PL (Shimadzu RF-5301PC, Japan), and the excitation wavelength was 322 nm.

### Adsorption Properties of the Material

50 ml of 20 mg/L TC solution was added into the beaker, then 20 mg of Fe-C_3_N_4_/Ti_3_C_2_ powder was added, and shook well. The mixture was stirred away from light, the proper amount of mixture was absorbed every 10 min, and the absorbance was measured at 356 nm by using a visible spectrophotometer.

### Photocatalytic Performance of the Material

50 ml of 20 mg/L TC solution was added into the beaker, 20 mg of Fe-C_3_N_4_/Ti_3_C_2_ powder was added, and shook well. The mixture was stirred away from light until the adsorption–desorption is balanced, then placed it in a xenon lamp source (*λ* > 420 nm, 280W), the proper amount of mixture was absorbed every 20 min, and the absorbance was measured at 356 nm by using a visible spectrophotometer. Benzoquinone (BQ), isopropyl alcohol (IPA), and ethylenediamine tetraacetic acid disodium (EDTA-2Na) were used as the trapping agent of superoxide free radical (•O_2_^−^), hydroxyl free radical (•OH), and hole (h^+^), respectively, to explore the degradation mechanism of pollutants in the photocatalytic reaction.

### Photoelectrochemical Measurements

The photoelectrochemical measurements were conducted on a three-electrode electrochemical workstation (A4602, Wuhan Kesite Instrument Co., LTD.), in which the working electrode, counter electrode, and reference electrode were the obtained samples, platinum foil, and Ag/AgCl electrode, respectively. The working electrodes were prepared by the drop-coating method: typically, 2 mg of catalyst and 15 μl Nafion solution were dispersed in 1 ml of ethanol and sonicated for 30 min. Afterward, the resulted homogeneous suspension was dripped onto the FTO and dried at room temperature. Here, all photoelectrochemical measurements were carried out in 0.5 m Na_2_SO_4_ electrolyte, and the light source was identical to that of photocatalytic measurements. Mott–Schottky was recorded at a frequency of 1,000 Hz.

## Results and Discussion

### XRD Characterization

**FIGURE 1:**
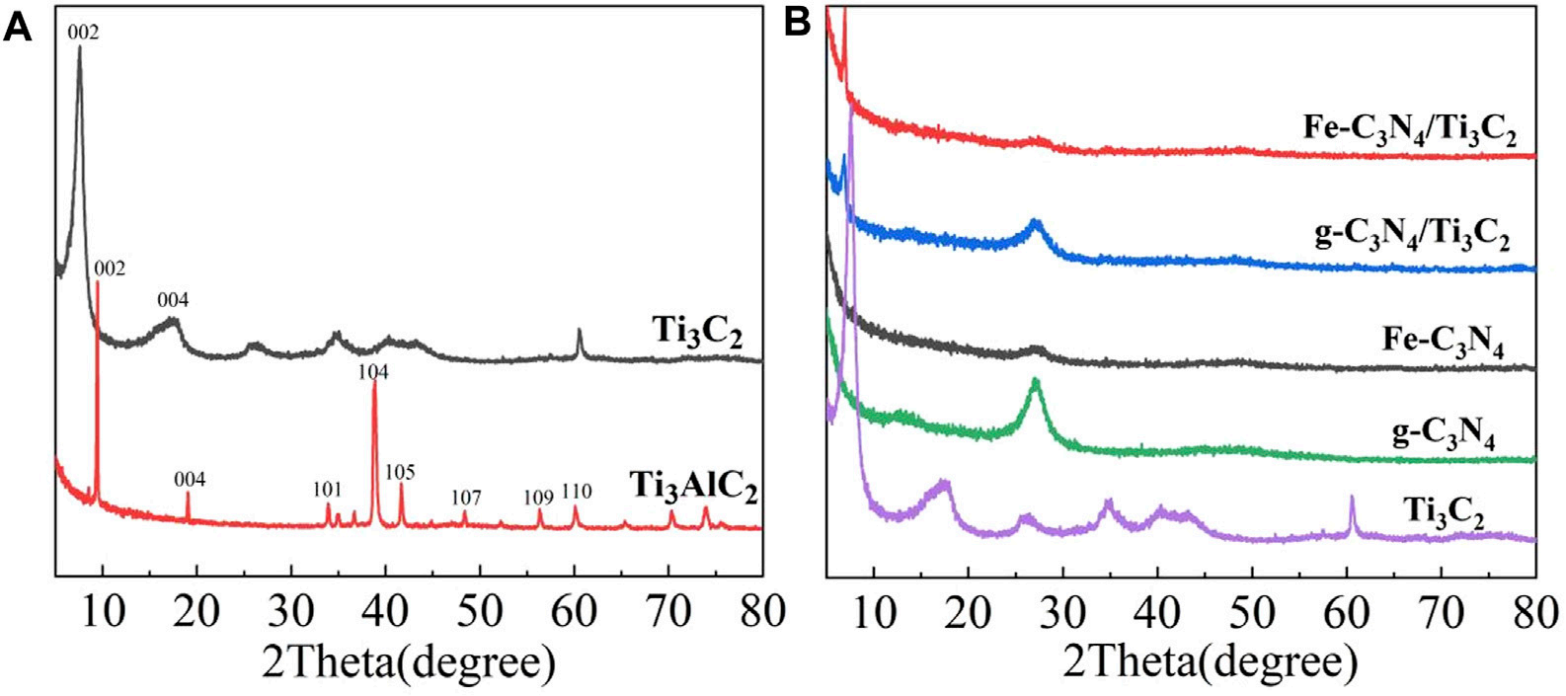
**(A)** XRD pattern of Ti_3_C_2_ and Ti_3_AlC_2_. **(B)** XRD pattern of the Ti_3_C_2_, g-C_3_N_4_, Fe-C_3_N_4_, g-C_3_N_4_/Ti_3_C_2,_ and Fe-C_3_N_4_/Ti_3_C_2_.

The phase of the sample was characterized by an X-ray powder diffractometer, and the results are reflected in Figure 1A which shows the XRD patterns of Ti_3_AlC_2_ and multilayer Ti_3_C_2_. A series of diffraction peaks corresponding to (002), (004), (101), (104), (105), (107), (109), and (110) crystal planes can be observed from the XRD spectra of Ti_3_AlC_2_. After being etched by HF, the strongest peak of Ti_3_C_2_ at 39.0° disappeared, and the peaks corresponding to (002) and (004) crystal planes widened and shifted to a lower angle, indicating that the Al layer in Ti_3_AlC_2_ had been removed, and Ti_3_AlC_2_ was successfully converted to Ti_3_C_2_. At the same time, the Ti_3_C_2_ layer structure becomes thinner. From Figure 1B, two evident diffraction peaks located at 12.83° and 27.05° for g-C_3_N_4_ are observed, corresponding to (100) and (002) crystal planes, respectively. Fe-C_3_N_4_ mainly had a peak at 27.05°, corresponding to the (002) crystal plane of g-C_3_N_4_. The characteristic peak of Fe was not observed, probably due to the small amount of doped Fe or the weak characteristic peak. The XRD pattern of g-C_3_N_4_/Ti_3_C_2_ shows two distinct peaks at 6.93° and 27.05°, respectively, corresponding to the (002) crystal plane of Ti_3_C_2_ and the (002) crystal plane of g-C_3_N_4_, indicating that g-C_3_N_4_ and Ti_3_C_2_ have achieved good recombination. The XRD pattern of Fe-C_3_N_4_/Ti_3_C_2_ is similar to that of g-C_3_N_4_/Ti_3_C_2_. The characteristic peaks of Ti_3_C_2_ and g-C_3_N_4_ are observed, and the characteristic peaks of Fe are not observed, on account of the small amount of Fe doped or the weak characteristic peaks.

Compared with multilayer Ti_3_C_2_, the intensity of characteristic peaks on the (002) plane of g-C_3_N_4_/Ti_3_C_2_ and Fe-C_3_N_4_/Ti_3_C_2_ at 6.93° is much weaker. It is speculated that NH_3_ released during urea calcination can be inserted into the interlayer of multilayer Ti_3_C_2_ as a small molecule in the process of compounding with Ti_3_C_2_, which effectively prevents the stacking of Ti_3_C_2_ interlayers, plays a supporting role, promotes the expansion of interlayer spacing of multilayer Ti_3_C_2_, and forms a close 2D/2D interlayer contact surface with g-C_3_N_4_. At the same time, it is speculated that Fe doping causes different degrees of polycondensation of g-C_3_N_4_ and delays the phase transition process, resulting in the decrease of cell parameters and crystal plane spacing of g-C_3_N_4_ and the increase of specific surface area. Moreover, Ti_3_C_2_ and g-C_3_N_4_ form a 2D/2D contact surface, which directly affects the periodic stacking interlayer structure of g-C_3_N_4_, resulting in the decrease of the diffraction peak intensity of g-C_3_N_4_ at 27.05° and the broadening.

### SEM Characterization

**FIGURE 2:**
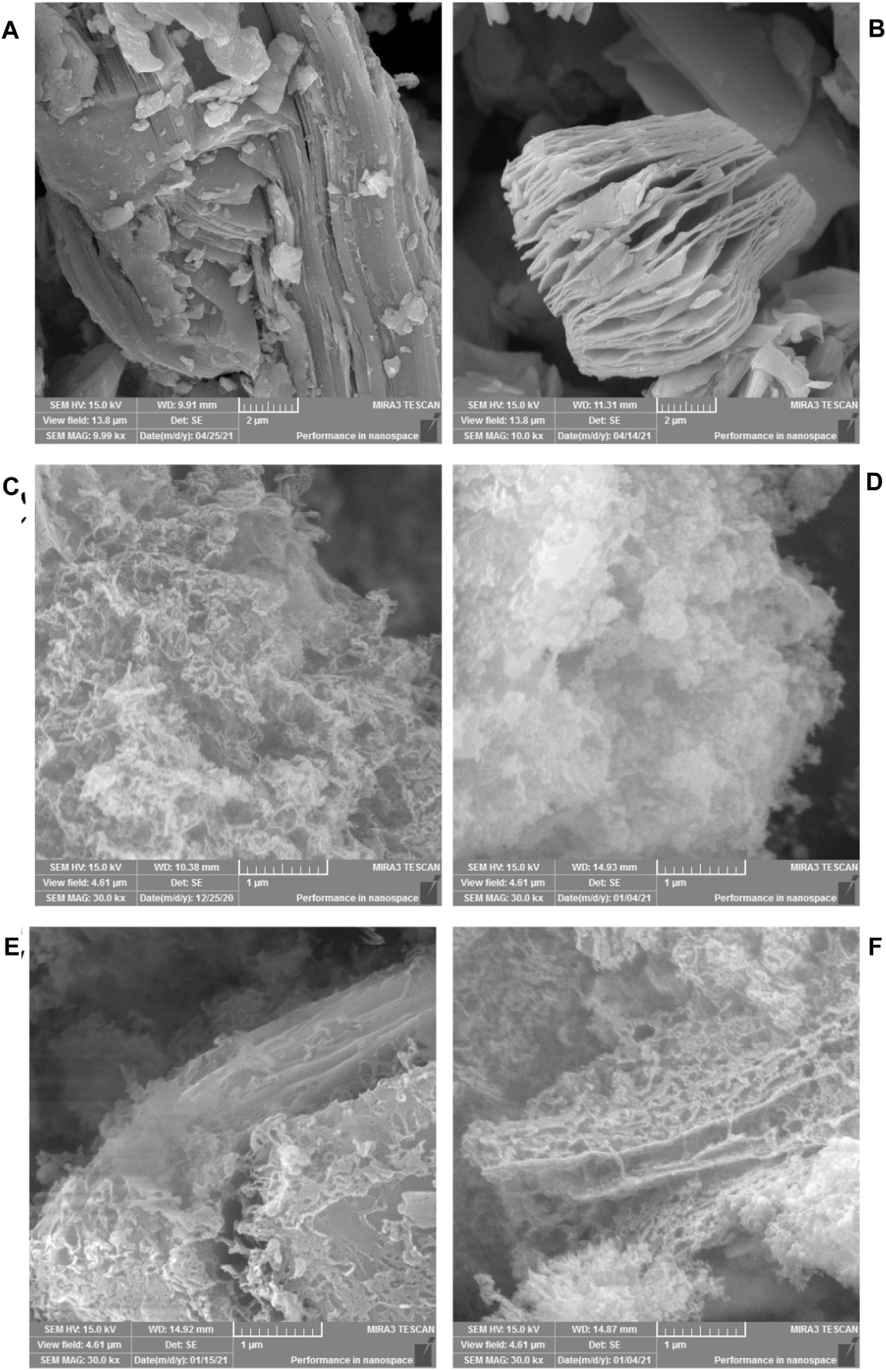
SEM images of **(A)** Ti_3_AlC_2_, **(B)** Ti_3_C_2_, **(C)** g-C_3_N_4_, **(D)** Fe-C_3_N_4_, **(E)** g-C_3_N_4_/Ti_3_C_2_ and **(F)** Fe-C_3_N_4_/Ti_3_C_2_.

The microstructure of the samples was characterized by SEM, and the results are shown in Figure 2. It can be seen from Figure 2A that the unetched Ti_3_AlC_2_ is a bulk stacking structure. The morphology of Ti_3_C_2_ obtained by etching is shown in Figure 2B. Clear interlamellar spacing can be observed visually, showing a two-dimensional layered structure of MXene. Multilayer Ti_3_C_2_ stacked together changed into an accordion-like structure. It is observed from Figure 2C that g-C_3_N_4_ is mostly agglomerated and a small part is thin. It is observed from Figure 2D that the aggregation of g-C_3_N_4_ after Fe-doping is weakened, showing a thin lamellar structure. It is observed from Figure 2E that, after the combination of g-C_3_N_4_ and Ti_3_C_2_, the two form a close interlayer structure. From Figure 2F, it can be observed that there is a close interlayer structure between g-C_3_N_4_ and Ti_3_C_2_ in Fe-C_3_N_4_/Ti_3_C_2_. It is assumed that urea releases NH_3_ during the calcination, and small molecule NH_3_ inserts into the interlayer structure of Ti_3_C_2_ and acts as a gas template to strip Ti_3_C_2_, effectively preventing the accumulation of Ti_3_C_2_ layers (Yang et al., 2020).

### TEM Characterization

**FIGURE 3:**
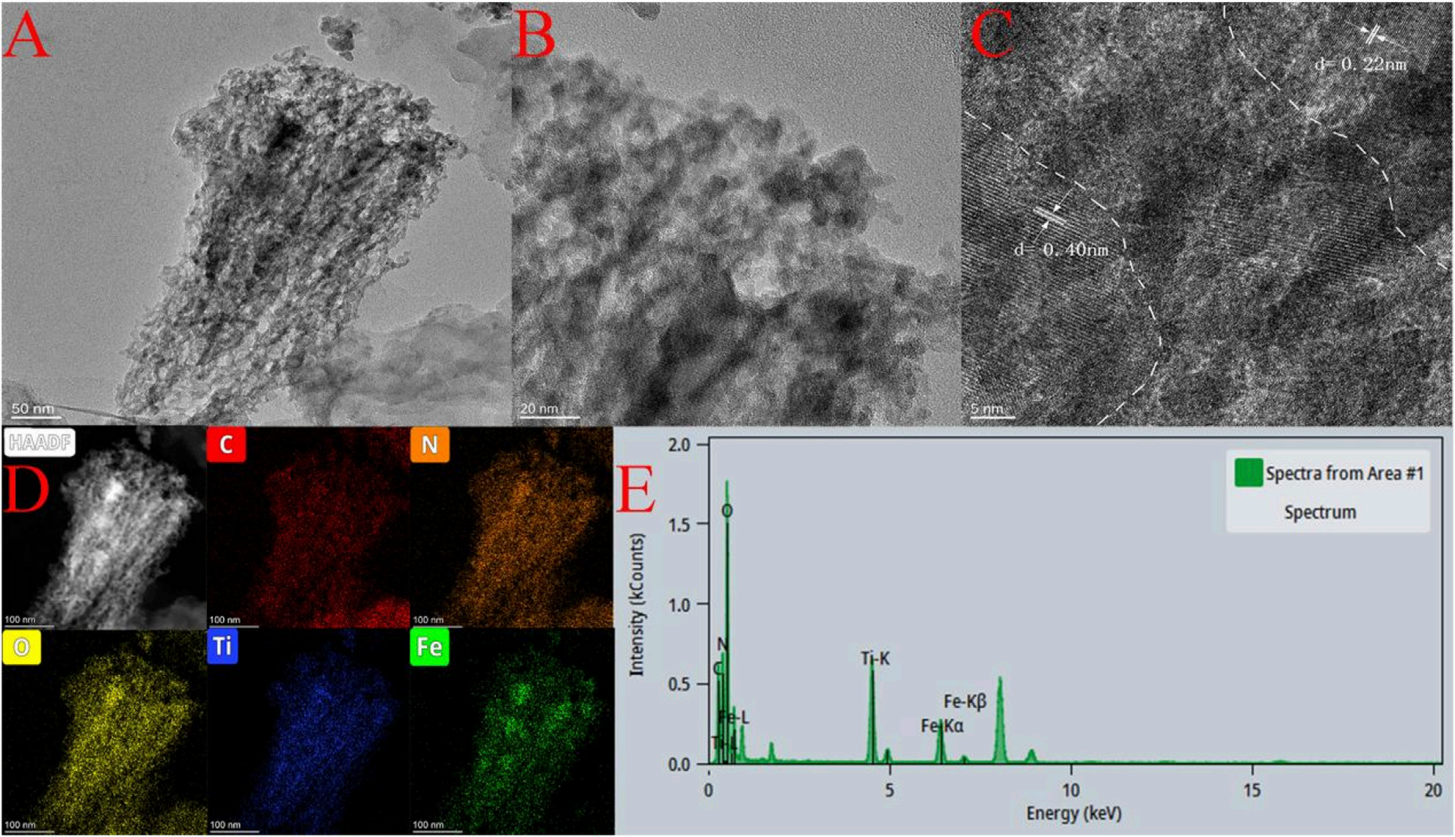
**(A,B)** TEM images of Fe-C_3_N_4_/Ti_3_C_2_, **(C)** HR-TEM of Fe-C_3_N_4_/Ti_3_C_2,_ **(D,E)** EDS of Fe-C_3_N_4_/Ti_3_C_2_.

The morphology and microstructure of the Fe-C_3_N_4_/Ti_3_C_2_ catalyst were observed by TEM (Figure 3A). It can be seen that the sample exhibits a typical layered structure. In Figure 3B, the shallowest area is g-C_3_N_4_, the darker overlying layer is Ti_3_C_2_, and the darkest black area is Fe species. More information about Fe-C_3_N_4_/Ti_3_C_2_ can be obtained from Figure 3C. The lattice spacing of Ti_3_C_2_ is 0.22 nm, and the lattice spacing of Fe species is 0.40 nm. In addition, the EDS element map (Figure 3D) of the sample further confirms that there is good interaction between Fe, Ti_3_C_2_, and g-C_3_N_4_. The elements C, N, O, Ti, and Fe are distributed uniformly in the samples. According to Figure 3E, the contents of Fe and Ti are 13.34 and 25.16%, respectively. These results show that g-C_3_N_4_ is uniformly modified by Ti_3_C_2_ and Fe and has close contact.

### XPS Characterization

**FIGURE 4:**
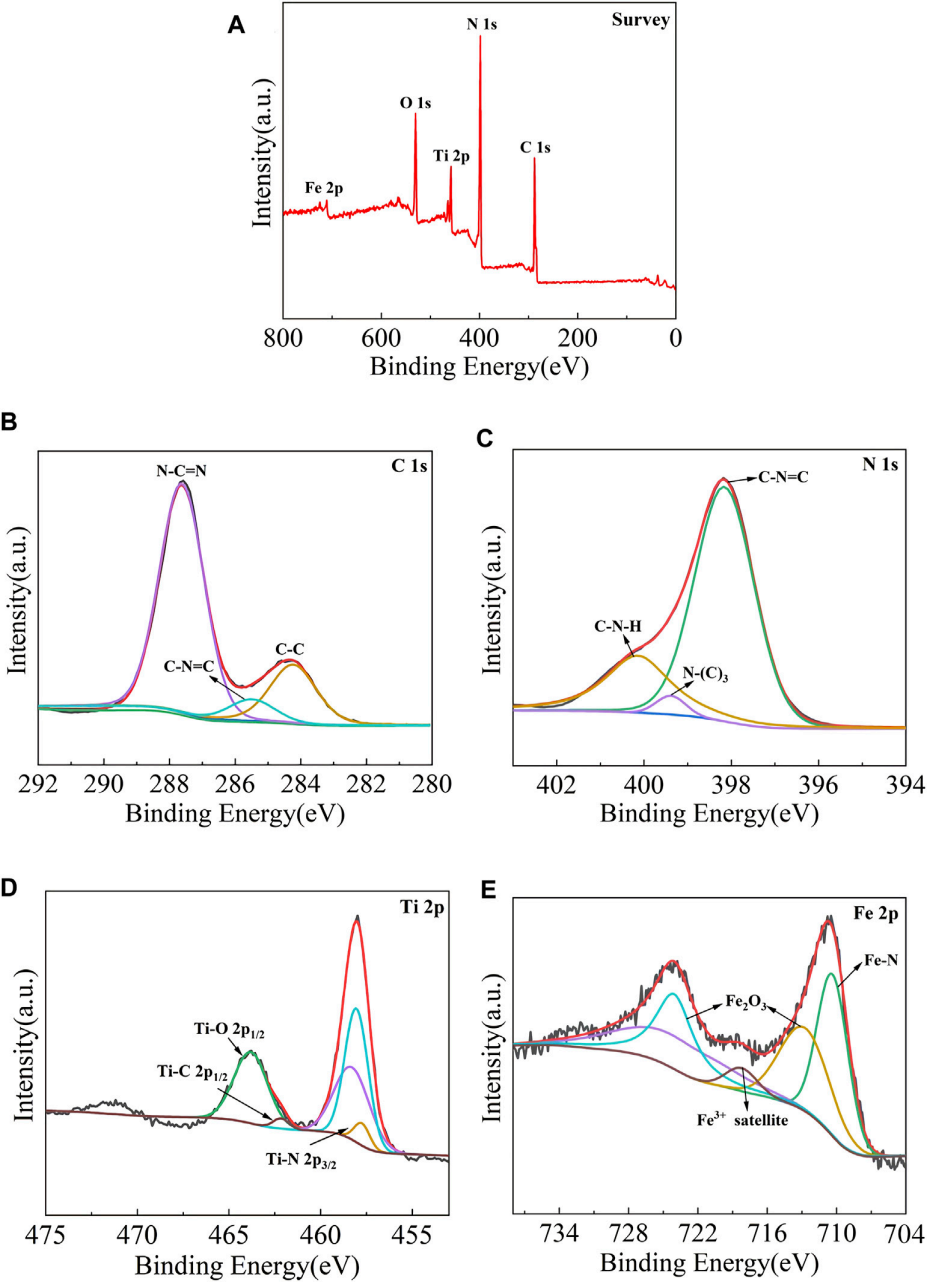
XPS pattern of **(A)** Fe-C_3_N_4_/Ti_3_C_2_ composites, High-resolution XPS spectra comparison of **(B,C)** 1s and **(C)** N 1s and **(D)** Ti 2pand **(E)** Fe 2p.

The chemical composition and valence state of the samples were characterized by X-ray photoelectron spectroscopy, and the results are shown in Figure 4. It is observed from Figure 4A that the measured spectra of Fe-C_3_N_4_/Ti_3_C_2_ show the existence of C, N, Ti, O, and Fe elements, indicating that Fe-doping and the composite of Ti_3_C_2_ were successfully realized in g-C_3_N_4_. It can be seen from the C 1s spectrum (Figure 4B) that the C 1s peak can be fitted into three peaks, namely, graphite phase carbon (C–C) at 284.38 eV, sp^3^-hybridized carbon (C-N=C) at 285.48 eV, and sp^2^-hybridized carbon (N-C=N) at 287.58 eV. The N 1s spectrum (Figure 4C) shows three distinct peaks, which are ascribed to sp^2^-hybridized nitrogen (C-N=C) at 398.18 eV, tertiary nitrogen group (N-(C)_3_) at 399.38 eV, and free amino groups (C-N-H) at 400.10 eV, respectively. According to the Ti 2p spectrum (Figure 4D), Ti 2p XPS peaks are deconvoluted into three peaks, namely, Ti-N bond at 457.70 eV, Ti-C 2p^1/2^ bond at 462.20 eV, and Ti-O 2p^1/2^ bond at 463.84 eV. The existence of the Ti-O 2p^1/2^ bond may be related to the slight oxidation of Ti_3_C_2_. According to the Fe 2p spectra (Figure 4E), there are three chemical states of Fe, namely, Fe-N bond at 710.48 eV, trivalent iron satellite peak at 718.30 eV, and trivalent iron in Fe_2_O_3_ at 713.08 and 724.28 eV, which indicates that there are both iron–nitrogen chemical bonds and Fe_2_O_3_ in the material. From the above analysis, Fe-doping and Ti_3_C_2_ composite were successfully realized in g-C_3_N_4_. In Fe-C_3_N_4_/Ti_3_C_2_, Fe mainly exists in the form of the Fe-N bond and Fe_2_O_3_, Ti forms the key existence in the Ti-N bond and Ti-C bond, which further confirms that Fe and Ti_3_C_2_ have been successfully combined with g-C_3_N_4_.

### TGA Characterization

**FIGURE 5:**
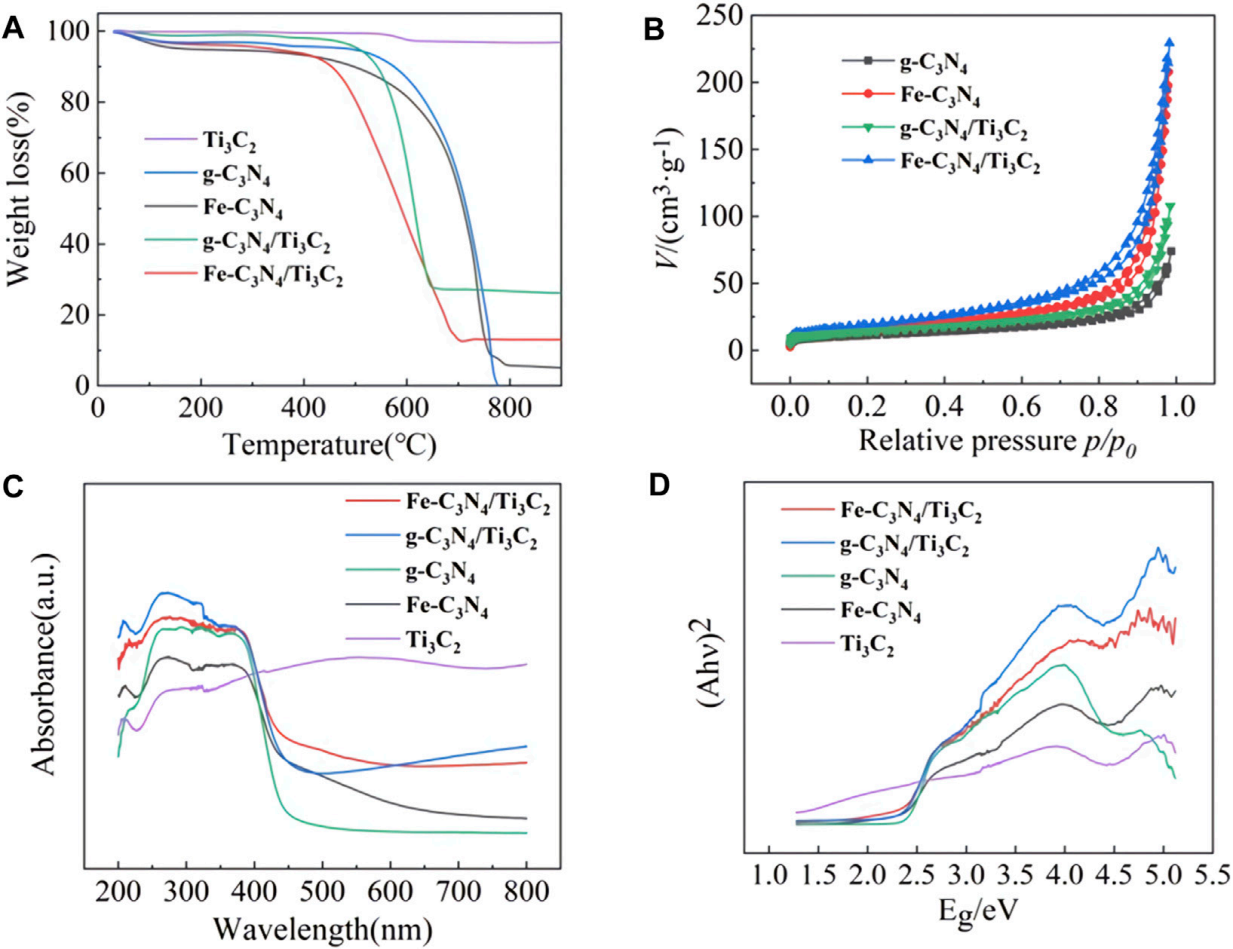
**(A)** The TGA curves of Ti_3_C_2_, g-C_3_N_4_, Fe-C_3_N_4_, g-C_3_N_4_/Ti_3_C_2,_ and Fe-C_3_N_4_/Ti_3_C_2._ **(B)** N_2_ adsorption-desorption isotherms of different materials. **(C)** UV-Vis diffuse reflectance spectra and **(D)** band gap of g-C_3_N_4_, Ti_3_C_2_, Fe-C_3_N_4_, g-C_3_N_4_/Ti_3_C_2_ and Fe-C_3_N_4_/Ti_3_C_2_.

The thermal stability of the sample was characterized by a thermogravimetric analyzer, and the results are shown in Figure 5A. It can be observed from Figure 5A that the mass change of Ti_3_C_2_ is not obvious with the increase in temperature, showing excellent stability. The mass loss of g-C_3_N_4_, Fe-C_3_N_4_, g-C_3_N_4_/Ti_3_C_2_, and Fe-C_3_N_4_/Ti_3_C_2_ was very rapid within a certain temperature range, which was due to the decomposition of the C-N bond of g-C_3_N_4_ in the material to generate CO_2_ and NO_2_. At the same time, pure g-C_3_N_4_ was completely decomposed when the temperature exceeded 777°C, while Fe-C_3_N_4_, g-C_3_N_4_/Ti_3_C_2_, and Fe-C_3_N_4_/Ti_3_C_2_ were not completely decomposed, which was attributed to the stability of Fe and Ti_3_C_2_ residues in the sample.

### BET Characterization

The specific surface area of the sample was analyzed by an automatic surface adsorption instrument. Figure 5B shows that the specific surface areas of g-C_3_N_4_, Fe-C_3_N_4_, g-C_3_N_4_/Ti_3_C_2_, and Fe-C_3_N_4_/Ti_3_C_2_ are 38.55 m^2^/g, 54.13 m^2^/g, 49.25 m^2^/g, and 68.58 m^2^/g, respectively. Both Fe-doping and composite Ti_3_C_2_ are conducive to expanding the specific surface area of photocatalyst, providing more active sites and improving the adsorption performance of materials. At the same time, the larger the specific surface area, the e^−^ generated by g-C_3_N_4_ can migrate to the surface of the catalyst more quickly, which effectively reduces the recombination rate of electron and hole pairs and improves the photocatalytic performance of the material.

### UV-Vis-DRS Characterization

The absorption properties of the samples were recorded by UV-Vis diffuse reflectance spectroscopy. The results are shown in Figures 5C,D. It is observed from Figure 5C that the Ti_3_C_2_ shows broad absorption in the whole region (200∼800 nm), and there is no obvious absorption with the edge, indicating that Ti_3_C_2_ has metallic properties. The pristine g-C_3_N_4_ displays a typical absorption region at about 450 nm, which is consistent with the literature (Gebreslassie et al., 2019). The optical cutoff wavelengths of Fe-C_3_N_4_, g-C_3_N_4_/Ti_3_C_2_, and Fe-C_3_N_4_/Ti_3_C_2_ are 467 nm, 458, and 470 nm, respectively. It can be seen that after Fe-doping and Ti_3_C_2_ composite, the sample shows a redshift compared with the monomer g-C_3_N_4_, and the visible light absorption capacity is enhanced, indicating that Fe-doping and composite Ti_3_C_2_ can effectively improve the visible light absorption performance of g-C_3_N_4_. Due to the strong absorption of dark Ti_3_C_2_ in the whole wavelength range (200∼800 nm), the absorption edge of g-C_3_N_4_ can readily shift after recombination, so Ti_3_C_2_ composite can effectively enhance the light absorption of g-C_3_N_4_. According to the energy level theory, it is suggested that the bandgap of g-C_3_N_4_ forms the impurity energy level after Fe-doping. The electrons only need to absorb photons with small energy to realize the indirect transition of energy level, which can absorb photons with long-wavelength, broaden the visible light absorption range of g-C_3_N_4_ and improve the utilization rate of visible light. From Figure 5D the band gaps of g-C_3_N_4_, Fe-C_3_N_4_, g-C_3_N_4_/Ti_3_C_2_, and Fe-C_3_N_4_/Ti_3_C_2_ are 2.38, 2.26, 2.21, and 2.19 eV, respectively. It shows that Fe-doping and composite Ti_3_C_2_ can reduce the bandgap energy of g-C_3_N_4_, reduce the bandgap width, expand the visible light response range, and improve the visible light utilization.

### PL Characterization

**FIGURE 6:**
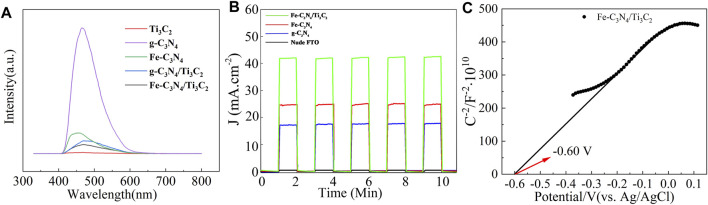
**(A)** PL spectra of g-C_3_N_4_, Ti_3_C_2_, Fe-C_3_N_4_, g-C_3_N_4_/Ti_3_C_2,_ and Fe-C_3_N_4_/Ti_3_C_2._ **(B)** The transient photocurrent density of nude FTO, g-C_3_N_4_, Fe-C_3_N_4_ and Fe-C_3_N_4_/Ti_3_C_2._ **(C)** Mott-Schottky curves of Fe-C_3_N_4_/Ti_3_C_2._

The photocatalytic activity of the samples was analyzed by fluorescence spectroscopy. The results are shown in Figure 6A. It can be seen from Figure 6A that g-C_3_N_4_ shows a strong fluorescence emission peak at 466 nm. After Fe-doping and composite Ti_3_C_2_, the intensity of this peak was inhibited significantly. Confirming that Fe-doping and composite Ti_3_C_2_ can effectively reduce the electrons and holes recombination probability of g-C_3_N_4_, and the quantum efficiency is improved.

According to the relevant literature (Li et al., 2008), Fe^2+^/Fe^3+^ has a reduction potential lower than the conduction band potential of g-C_3_N_4_, so Fe-doping can effectively capture the photogenerated carriers of g-C_3_N_4_ and inhibit the recombination of electron and hole pairs. When the g-C_3_N_4_ layer is inserted into the Ti_3_C_2_ sheet, the two form many intimate interfaces, thus providing the maximum contact surface between the two phases. On such a platform, electrons can be easily transferred from g-C_3_N_4_ nanosheets to metal Ti_3_C_2_ sheets with high conductivity (Su et al., 2019), inhibiting the recombination of electron and hole pairs. At the same time, it can be observed from Figure 6A that the fluorescence intensity of Fe-C_3_N_4_/Ti_3_C_2_ is the lowest, indicating that there is a synergistic effect between Fe-doping and composite Ti_3_C_2_ in g-C_3_N_4_. The recombination rate of the excited electrons and holes in g-C_3_N_4_ is greatly reduced, the quantum efficiency shows excellent improvement, and the sample may show better photocatalytic activity.

As it is known to all, a higher photocurrent means a higher separation efficiency for the photogenerated charge, which is finally reflected in the better photocatalytic performance. As displayed in Figure 6B, compared with naked FTO, the photocurrent responses of g-C_3_N_4_, Fe-C_3_N_4_, and Fe-C_3_N_4_/Ti_3_C_2_ samples gradually increase, with average photocurrent densities of 17.3, 24.6, and 41.7 μA/cm^2^. Fe-C_3_N_4_/Ti_3_C_2_ shows the highest photocurrent density, which is 2.4-fold higher than g-C_3_N_4_ and 1.7-fold higher than Fe-C_3_N_4_. The enhanced photocurrent density of Fe-C_3_N_4_ and Fe-C_3_N_4_/Ti_3_C_2_ electrodes, respectively, shows that Fe doping and Ti_3_C_2_ composite can promote g-C_3_N_4_ to provide more carriers to the external circuit, resulting in the greater the photocurrent and the higher the photogenerated charge. The higher the separation efficiency, the better the photocatalytic performance. The results here are mainly due to the following reasons: the reduction potential of Fe^2+^/Fe^3+^ is lower than the conduction band potential of g-C_3_N_4_, so Fe can effectively capture the photogenerated carriers generated by g-C_3_N_4_, and the e^−^–h^+^ pair recombination rate is greatly reduced (Hu et al., 2018), and Fe doping weakens the degree of polymerization of g-C_3_N_4_, increases its surface area, and provides more photocatalytically active sites. The Ti_3_C_2_ layer is embedded in thin g-C_3_N_4_ nanosheets, forming a heterojunction and interfacial effect between them. The photogenerated electrons tend to transfer from g-C_3_N_4_ to Ti_3_C_2_, which improves the interfacial charge transfer ability of g-C_3_N_4_, which can effectively inhibit the recombination of e^−^–h^+^ pairs. (Li et al., 2020). Metal Fe-doped and g-C_3_N_4_ composite Ti_3_C_2_ act simultaneously, and the photocatalytic performance is better than Fe-C_3_N_4_.

### Adsorption Performance

**FIGURE 7:**
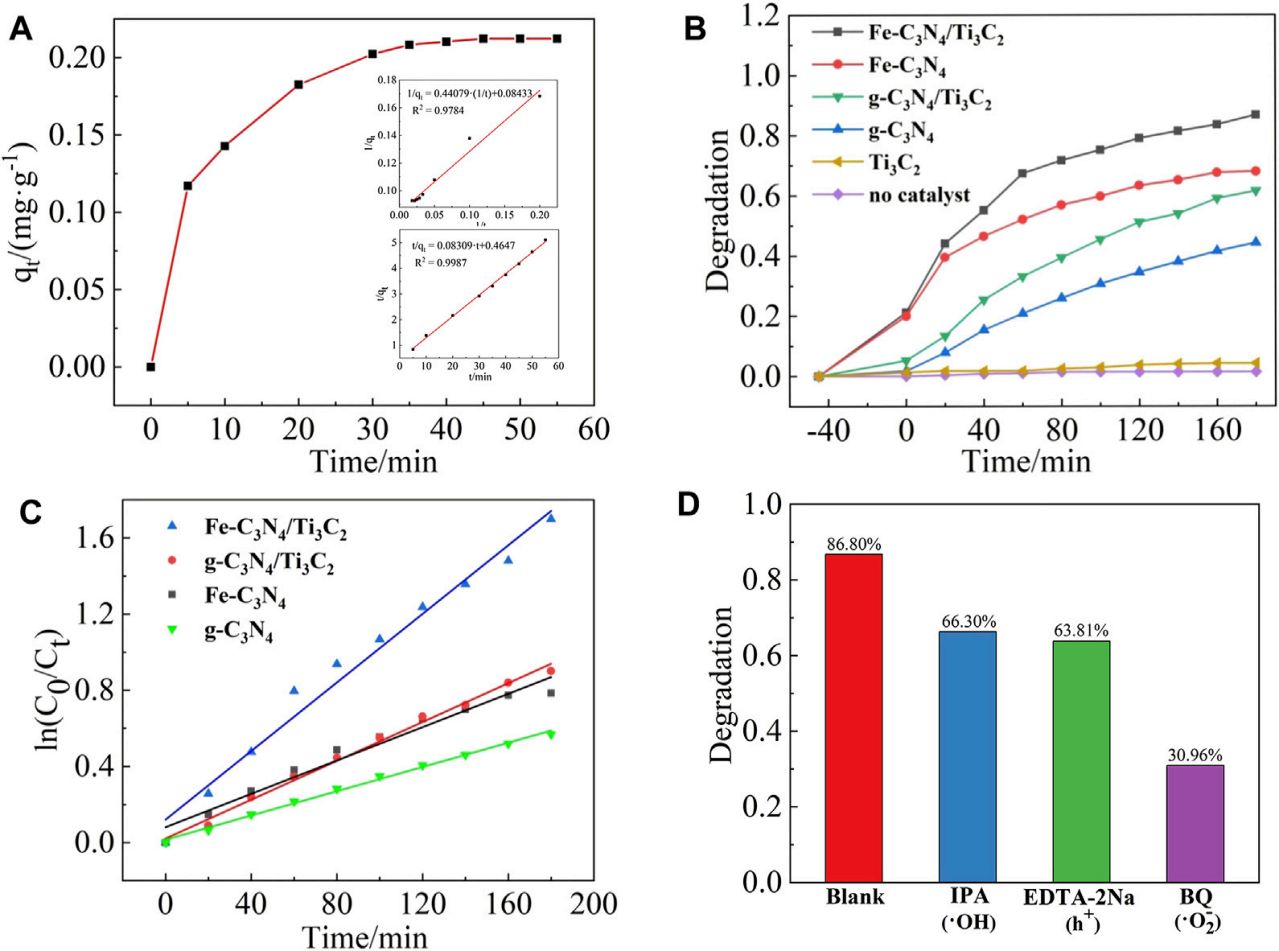
**(A)** Absorption curve of TC on Fe-C_3_N_4_/Ti_3_C_2。_Dynamic model: quasi-first order,quasi-second order. **(B)** Photocatalytic degradation curves of TC by different photocatalysts. **(C)** Kinetic fitting curve. **(D)** Photocatalytic activity changes of Fe-C_3_N_4_/Ti_3_C_2_ for TC degradation with different sacrificial agents.

When the catalyst dosage was 20 mg and the initial concentration of TC is 20 mg/L, the adsorption curve of Fe-C_3_N_4_/Ti_3_C_2_ on TC is shown in Figure 7A. In the first 5 min of the dark reaction, the adsorption amount of TC on Fe-C_3_N_4_/Ti_3_C_2_ increased rapidly and reached the adsorption-desorption equilibrium after 45 min. The equilibrium adsorption amount was 10.78 mg/g, and the adsorption rate was 21.03%. The adsorption kinetics of TC on Fe-C_3_N_4_/Ti_3_C_2_ was investigated by pseudo-first-order and pseudo-second-order adsorption kinetics models. The results are shown in Figure 7A. The graph shows that the correlation coefficient of the pseudo-first-order kinetic equation *R* ^2^ = 0.9784, and the correlation coefficient of the pseudo-second-order kinetic equation *R* ^2^ = 0.9987, indicating that the adsorption of TC by Fe-C_3_N_4_/Ti_3_C_2_ is a second-order kinetic model, that is, chemical adsorption. The equilibrium adsorption amount *q*_*e*_ = 10.78 mg/g, and the adsorption rate constant *k* = 0.01852 g/(mgmin) can be obtained from the slope and intercept of the straight line, respectively.

### Photocatalytic Activity

When the catalyst dosage was 20 mg and the initial concentration of TC was 20 mg/L, the photocatalytic degradation curves of different samples are shown in Figure 7B. When no photocatalyst was added, TC was almost not degraded under xenon lamp irradiation, and the degradation rate was only about 1.41%, indicating that TC was relatively stable and difficult to be photodegraded. When only Ti_3_C_2_ was added, the photocatalytic degradation rate of TC under xenon lamp irradiation was only 4.30%, indicating that Ti_3_C_2_ cannot be used as a catalyst for photocatalytic degradation of TC alone. After 180 min of illumination, the photocatalytic degradation rates of TC by g-C_3_N_4_, g-C_3_N_4_/Ti_3_C_2_, Fe-C_3_N_4_, and Fe-C_3_N_4_/Ti_3_C_2_ were 44.37, 61.53, 68.01, and 86.80%, respectively. It can be seen that compared with g-C_3_N_4_, the degradation rate of TC by g-C_3_N_4_ is improved after Fe-doping and composite Ti_3_C_2_. The degradation rate of TC by Fe-C_3_N_4_/Ti_3_C_2_ within 180 min is 86.80%, 1.96 times that of g-C_3_N_4_, which greatly improves the photocatalytic degradation efficiency of TC by photocatalyst. It can be speculated that there is a synergistic effect between Fe-doping and composite Ti_3_C_2_ in g-C_3_N_4_, and the photocatalytic ability of the sample is better.

Linear fitting with $\ln\left(C_{0}/C_{t}\right)$ to *t*, and the results are shown in Figure 7C. The photocatalytic degradation of TC by Fe-C_3_N_4_/Ti_3_C_2_ conforms to the first-order reaction kinetics model, and the relevant parameters are shown in Table 1. The apparent rate constants k of Fe-C_3_N_4_, g-C_3_N_4_/Ti_3_C_2_, and Fe-C_3_N_4_/Ti_3_C_2_ are 1.38, 1.60, and 2.83 times of g-C_3_N_4_, respectively. It shows that Fe-doping and composite Ti_3_C_2_ are beneficial to improve the photocatalytic degradation activity of g-C_3_N_4_.

**TABLE 1:** First-order reaction kinetic parameters.

Photocatalyst	First order kinetic equation	Correlation R^2^	Degradation rate (%)	Reaction Rate Constant k (min)
g-C_3_N_4_	ln (C_0_/C_t_) = 0.00318t+0.01508	0.9957	44.37	0.00318^–1^
Fe-C_3_N_4_	ln (C_0_/C_t_) = 0.00438t+0.08071	0.9664	68.01	0.00438^–1^
g-C_3_N_4_/Ti_3_C_2_	ln (C_0_/C_t_) = 0.00510t+0.02109	0.9937	61.53	0.00510^–1^
Fe-C_3_N_4_/Ti_3_C_2_	ln (C_0_/C_t_) = 0.00901t+0.12033	0.9794	86.80	0.00901^–1^

### Degradation Mechanism

By adding sacrificial agents such as BQ, IPA, and EDTA-2Na to detect the common active radicals •O_2_^−^, •OH, and h^+^ to explore the degradation mechanism of the photocatalytic reaction system. The results are shown in Figure 7D. When no radical scavengers were in the solution, the degradation rate of TC by Fe-C_3_N_4_/Ti_3_C_2_ was 86.80%. When •OH and h^+^ capture agents were added, the photocatalytic degradation of TC by Fe-C_3_N_4_/Ti_3_C_2_ was inhibited to a certain extent, and the degradation rates were reduced to 66.30 and 63.81%, respectively. When •O_2_^−^ capture agent was added, the photocatalytic degradation of TC by Fe-C_3_N_4_/Ti_3_C_2_ was greatly inhibited, and the degradation rate decreased to 30.96%. Therefore, the influence of active species on the degradation of TC in the photocatalytic degradation system was as follows: •O_2_^−^ > h^+^ > •OH.

**FIGURE 8:**
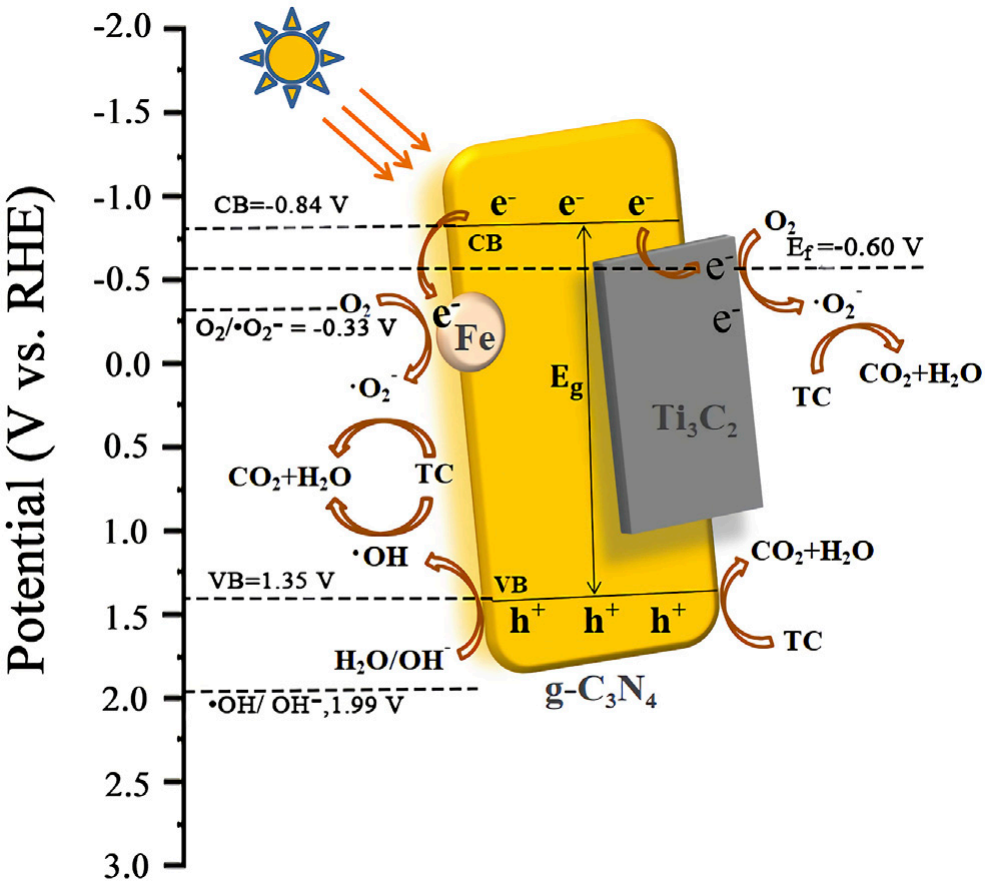
Photocatalytic degradation mechanism diagram of Fe-C_3_N_4_/Ti_3_C_2_ to TC.

Based on the above results, we speculated on the photocatalytic degradation mechanism of Fe-C_3_N_4_/Ti_3_C_2_ to TC (Figure 8). The electrons of g-C_3_N_4_ in Fe-C_3_N_4_/Ti_3_C_2_ were excited to CB under visible light irradiation. Thus, the photogenerated holes (h^+^) were left in the VB. In the Mott–Schottky test (Figure 6C), we made a tangent along the longest straight line in the figure, and the slope was found to be positive, indicating that g-C_3_N_4_ was an *n*-type semiconductor. According to the formula (E_g_ = E_VB_ + E_CB_) and the DRS results, the theoretical estimation of the E_VB_ and E_CB_ of g-C_3_N_4_ were 1.35 V and −0.84 V vs. NHE. In addition, the Fermi level (E_f_, vs. NHE, pH = 0) of Ti_3_C_2_ was −0.60 V, which was higher than the E_CB_ of g-C_3_N_4_ (Su et al., 2019). In Fe-C_3_N_4_/Ti_3_C_2_ composites, a close 2D/2D contact interface is formed between Ti_3_C_2_ and g-C_3_N_4_. Electrons could be easily transferred from g-C_3_N_4_ nanosheets to Ti_3_C_2_ surfaces, which inhibits recombination of photoinduced electrons and holes. Moreover, because the reduction potential of Fe^2+^/Fe^3+^ (Fe^2+^/Fe^3+^ = −0.77 V) was lower than the E_CB_ of g-C_3_N_4_ (Li et al., 2008), Fe could effectively capture the photogenerated electrons of g-C_3_N_4_ and inhibit recombination of electron and hole pairs. Then, the electrons reacted with oxygen adsorbed on the surface of the photocatalyst or dissolved in solution to form strongly oxidized •O_2_^−^ (O_2_/•O_2_^−^ = −0.33 V). It oxidized TC to CO_2_ and H_2_O. At the same time, the h^+^ of g-C_3_N_4_ reacted with H_2_O or OH^−^ in the solution to form the •OH (•OH/OH^−^ = 1.99 V) with strong oxidizability. •OH could degrade TC into CO_2_ and H_2_O. The holes in the valence band of g-C_3_N_4_ also had strong oxidation which could react with TC and degrade it. The process of Fe-C_3_N_4_/Ti_3_C_2_ photocatalytic degradation of TC is as follows.

### Material Performance Comparison

Comparing the experimental system with other related systems, the results are shown in Table 2. It can be seen from Table 2 that the experimental system has a certain value in practicability and economy. A small amount of Fe-doping and a small amount of Ti_3_C_2_ composite were used to modify g-C_3_N_4_, and the prepared Fe-C_3_N_4_/Ti_3_C_2_ photocatalyst could be used to degrade TC efficiently with a degradation rate of 86.80%. Compared with the following table, there are certain differences in the degradation rates of different substances in different systems. The main reasons are that the types and ratios of materials, the dosage of photocatalyst, the degradation time, and the power and wavelength of the light source are different.

**TABLE 2:** Comparison of photocatalytic properties of different systems.

Years	Photocatalyst	Dosage (mg)	Target degradation amount	Degrading time (min)	Source power (W)	Light source wavelength	Degradation rate (%)	Rate constant	References
2021	Fe-C_3_N_4_/Ti_3_C_2_	20	TC	180	280	>420 nm	86.80	0.0090 min^−1^	Our work
2021	Ti_3_C_2_/g-C_3_N_4_	20	LEV	30	300	>420 nm	72.00	0.0392 min^−1^	Liu et al. (2021)
2021	cPTA/g-C_3_N_4_	30	TC	180	500	>420 nm	78.00	0.0084 min^−1^	Yang et al. (2021)
2020	Ti_3_C_2_/alkalized g-C_3_N_4_	10	TC	60	300	>420 nm	77.00	0.0307 min^−1^	Yi et al. (2020)
2020	Fe-doped g-C_3_N_4_	50	RhB	60	300	>420 nm	87.00	-	Liu et al. (2020a)
2018	Fe-doped alkalinized g-C_3_N_4_	30	TC	80	300	>420 nm	63.70	0.0164 min^−1^	Xu et al. (2018)

## Conclusion

In this study, Fe-C_3_N_4_/Ti_3_C_2_ photocatalyst was synthesized *via* one-pot microwave method and high-temperature calcination method. When the catalyst dosage was 20 mg and the initial concentration of TC was 20 mg/L, the degradation rate of TC by Fe-C_3_N_4_/Ti_3_C_2_ was 86.80%, which was 1.96, 1.28, and 1.41 times that of g-C_3_N_4_, Fe-C_3_N_4_ and g-C_3_N_4_/Ti_3_C_2_. The influence of active species on the degradation of TC in the photocatalytic degradation system was •O_2_^−^ > h^+^ > •OH. During the synthesis of Fe-C_3_N_4_/Ti_3_C_2,_ the byproduct NH_3_ could intercalate multilayer Ti_3_C_2_, effectively preventing the stacking of Ti_3_C_2_ layers. Fe-doping and the composite of Ti_3_C_2_ would decrease the bandgap energy of g-C_3_N_4_ and effectively inhibited the recombination of electron and hole pairs of g-C_3_N_4_. It is expected that this work provides new insight into the construction of 2D/2D heterojunction materials used in the photocatalytic application.

## Data Availability

The original contributions presented in the study are included in the article/Supplementary Material, further inquiries can be directed to the corresponding author.
